# The associations of use of social network sites with perceived social support and loneliness

**DOI:** 10.1007/s12144-021-02673-9

**Published:** 2022-01-27

**Authors:** Vanessa Caba Machado, David Mcilroy, Francisca M. Padilla Adamuz, Rebecca Murphy, Susan Palmer-Conn

**Affiliations:** 1 Faculty of Health. School of Psycholoy, Liverpool John Moores University, Byrom Street, Liverpool, L3 3AF, Merseyside, England, UK; 2 Department of Experimental Psychology, University of Granada, Campus Universitario de Cartuja, 18071 Granada, Spain; 3 School of Sport and Exercise Sciences, Liverpool John Moores University, Byrom Street, Liverpool, L3 3AF, Merseyside, England, UK

**Keywords:** Social network sites (SNS), perceived social support, loneliness

## Abstract

Research shows that use of social network sites is associated with loneliness and this may be amplified in tertiary students by their transition from home life, especially if they struggle to integrate with peers. The buffering effects of social support may offer a solution and the online dimension may offer a suitable outlet for lonely and isolated students. In this study, *N* = 111 university students, aged 18-40, completed a frequency assessment of Instagram and WhatsApp, the Spanish version of the UCLA loneliness scale and the Multidimensional Scale of Social Support in an online survey. The statistical analysis was completed by Structural Equation Modeling using AMOS 25.0. The construct validity of social network sites was established by good factor loadings for WhatsApp and Instagram, but Facebook was excluded as it did not load adequately on to the latent measurement model, in keeping with the diminishing trend for Facebook use in young students. Loneliness emerged as pivotal in a mediation model, and online social support from friends/significant others, emerged as salient in the predictive model in contrast to family. However, these associations may not have the same advantageous weight for mature students given the observed negative associations with age. Results may have implications for policy and planning through highlighting the psychological variables that are operative in the dynamics of integration, retention, and adjustment to tertiary level experience.

## Introduction

Social media continues to grow, and human beings seem to be more connected to each other currently than ever before. Young people are continually online and connected to their social network sites (SNS), and they are the most avid users (Alzougool, 2018; Cohen et al., 2018). A study conducted by the Pew Research Center found that 91% of smartphones owners, aged 18-29, used SNS compared with 55% of those 50 and older (Smith, 2015). At the same time, the younger age group suffer the highest incidence rates of loneliness as reported in the literature (Griffin, 2010; Luhmann & Hawkley, 2016). Loneliness and lack of perceived social support are important factors for university students who face new challenges and may be geographically distant from home (Arnett et al., 2014; Diehl et al., 2018). Moreover, the focus on students is concordant with recent research. A study conducted by Razavi (2021) examined gender differences on the effect of the use of virtual social network sites on academic performance. Academic performance is an important life domain for the youth population, and it is also influential in judgments of well-being and overall life satisfaction. While the study found no gender differences, the author established the need for conducting more research examining the impact of SNS on other important aspects of students’ life such as mental health. This is of key importance, as Razavi (2013) established that academic performance implies the full repertoire of behavioral, metacognitive, cognitive, and motivational activities are implicated in students’ learning.

Researchers have been consistently attentive to loneliness and social support (Expósito & Moya, 1999; Lee et al., 2020; Perlman & Peplau, 1981). At the present time, the introduction of social media may have changed the dynamics of human relationships and this is affecting loneliness and social support in different ways.

The belief of available social support, which corresponds to the concept of perceived social support, is one of the most important factors concerning loneliness (Salimi & Bozorgpour, 2012). Loneliness and perceived social support have been shown to be negatively correlated in several studies (Salimi & Bozorgpour, 2012; Wang et al., 2018a), suggesting that those with high levels of loneliness may also have low levels of perceived social support (Cacioppo et al., 2006; Harrison et al., 2021a; Zhou et al., 2008). However, the research assessing the relationships between these two concepts and SNS use, is limited. Previous research has demonstrated a relationship between an active use of SNS and loneliness and the mediating role of perceived social support (Lin et al., 2020). Results from the study conducted by Lin et al. (2020) suggest that the relationship between active SNS usage and lower loneliness is explained by an increase in social support and self-esteem. Nevertheless, to our knowledge, the use of SNS in relation to perceived social support (with loneliness as a mediator) has not yet been investigated. This is a gap in this area of research, considering that in the relationships between social support and well-being, loneliness has been examined as a mediator by Stroebe et al. (1996). This finding raises the possibility that SNS usage affects perceived social support through levels of loneliness, and thus extend that old research in this new context.

Research has considered the relationships among specific SNS such as Facebook, perceived social support, and well-being (Lönnqvist & große Deters, 2016). However, conflicting results have been found in this research context (Lee et al., 2018). Research has shown that certain factors such as active or passive SNS usage (Lin et al., 2020; Liu et al., 2018), as well as strong ties and weak ties (Krämer et al., 2021) play an important role in the social support received and perceived. In fact, active usage and strong ties are more important in reducing loneliness and increasing social support than weak ties and passive usage (Krämer et al., 2021; Lin et al., 2020).

A research study conducted by Oshio et al. (2020) examined the mediating role of perceived social support in the relationship between SNS usage and satisfaction with life. These authors found that the use of SNS may increase life satisfaction through an increase in perceived social support. However, a limitation of their study is that they focused on the reported number of SNS friends as an indicator of SNS use, which might not be a valid indicator of an individual’s SNS usage. A narrative review conducted by Meng et al. (2017) examined research investigating social support and SNS. They identified 88 relevant articles, in which they found that Facebook was the most frequently studied platform. This is a limitation in the literature which leads to no generalizable results to more recent platforms (e.g., Instagram). Therefore, the current study will fill this gap in the literature focusing on other SNS platforms (Instagram and WhatsApp). Furthermore, the current study will measure self-reported frequency usage with the same scale developed by Rosen et al. (2013), which is considered a refined frequency measure.

### SNS Use and Perceived Social Support

This study is focused on perceived social support, or the individual’s perception of the availability of the support if needed. Research has demonstrated that perceived social support is equally important to received social support (Cohen et al., 2000). Nevertheless, moderate associations have been found between received support and perceived support in the literature. For instance, Haber et al. (2007) found a correlation of *r* = 0.35 between both types of social support.

Perceived social support is especially relevant in the context of university students as it is a protective factor against the high prevalence of stress, anxiety and depression symptoms that can be found in this population (Gallagher, 2008; Mackenzie et al., 2011; Price et al., 2006). Research has found repeatedly that perceived social support is a strong predictor of young people’s well-being (Chu et al., 2010; Nilsen et al., 2013; Wight et al., 2006). Furthermore, several studies have shown that perceived social support predicts a better psychological adjustment than offline received support and online received support (Li et al., 2015; Trepte et al., 2015; Trepte & Scharkow, 2016).

With regards to SNS use and social support, a meta-analysis conducted by Liu et al. (2018) concluded that general SNS use, and social support were moderately and positively correlated. In addition, they meta-analysed the data assessing various SNS activities, which showed different associations with social support. Findings showed that number of SNS friends and SNS self-presentation (activities of sharing photos and status updates) were positively and significantly associated with social support. Nevertheless, SNS interaction and content consumption were not significantly associated with social support. Overall, researchers support the conclusion that SNS facilitates the reception of both perceived and received social support (Lin et al., 2020; Su & Chan, 2017; Tifferet, 2020). However, the relationship between SNS and perceived social support is complicated and multifaceted (Liu et al., 2018). A study conducted by Oh et al. (2014) found that frequency of SNS use was not a significant predictor of perceived social support. This result may suggest that different sources of perceived social support could lead to different outcomes in the relationship between frequency of SNS use and this construct. In fact, regarding different sources of social support (e.g., family or friends) the relationship is still unknown. In conclusion, the impact of SNS on social support warrants more research attention (Li et al., 2015) because social support may be one of the major incentives for using SNS (Jung & Sundar, 2016; Kim et al., 2011).

### SNS and Loneliness

Loneliness corresponds to a divergence between one’s perceived and desired levels of social connectedness (Heinrich & Gullone, 2006). In addition, loneliness is a problem that all people experience worldwide to some extent and is a topic of interest for all age groups (Jeste et al., 2020; Nicolaisen & Thorsen, 2014). However, it is a paradox that in societies and age groups among which SNS are most widely used at its highest, people experience highest levels of loneliness (Pittman & Reich, 2016). In this new world of constant connectivity, a high proportion of the population feel detached from society in a maladaptive way. Therefore, this issue raises the question of why loneliness is so high in this population that is using SNS to stay connected more than any society in history.

Much work on the associations between SNS and loneliness has been carried out (Lin et al., 2020). A longitudinal study conducted by Brandtzæg (2012) showed a rise in loneliness in SNS users when compared to nonusers. Moreover, a more recent study conducted by Phu and Gow (2019) found that loneliness was predicted by persistence of Facebook usage (emotional connectedness towards the platform) but was not predicted by time spent on Facebook. However, results showed a negative association between number of Facebook friends and loneliness. On the other hand, Lin et al. (2020) found that an active usage of SNS is associated with lower levels of loneliness and that social support mediates this relationship. While this finding constitutes a contribution to our knowledge in this area of research, and helps to develop intervention programs, future research should explore the mediating role of loneliness.

Apart from mixed results found in the literature, there is even more disagreement regarding the direction of possible causal effect. Thus, researchers have been interested in whether SNS use makes people lonelier or whether lonely people tend to use their SNS platforms more frequently (Primack et al., 2017; Sampasa-Kanyinga & Lewis, 2015). For example, a meta-analytical study conducted by Song et al. (2014) suggests that excessive Facebook use is caused by loneliness rather than contrariwise. This meta-analysis showed that lack of social support activates loneliness, and that loneliness leads to Facebook use. Based on this conclusion, people use Facebook to seek social support when they may be lonely. However, this meta-analysis was only focused on Facebook use, and therefore, cannot be generalized to other platforms such as Instagram or WhatsApp. Moreover, Hunt et al. (2018) conducted an experimental study in which they found that reducing the time of social media usage for three weeks decreased the levels of loneliness. In addition, as mentioned previously, the evidence suggests that loneliness leads to decreased levels of perceived social support (Cacioppo et al., 2006; Zhou et al., 2008). Given the relationships found in the literature between SNS use, loneliness, and perceived social support, it is tentatively suggested that loneliness acts like a mediator in the relationship between SNS use and perceived social support. However, to the best of our knowledge, this mediation model of the relationship between SNS use and perceived social support, with the mediating role of loneliness, has not yet been investigated. Therefore, based on the evidence reported in the literature, this paper postulates loneliness as a likely mediator of SNS usage and social support relationship, which in turn aims to enhance the existing understanding of this research topic.

### Theoretical Approaches

There are three major theoretical and conceptual frameworks that have value in approaching this research topic. One theoretical perspective in the literature is Self-Determination Theory (SDT) (Ryan & Deci, 2000). This theory postulates three crucial psychological needs: autonomy, competence, and relatedness (Ryan & Deci, 2000). Specifically, research assessing the outcomes of SNS usage, has focused on the need for relatedness (Sheldon et al., 2011) for its relevance to the SNS context. Sheldon et al. (2011) found that the usage of an SNS platform, specifically Facebook, showed bidirectional outcomes. They found a positive correlation between Facebook usage and disconnection because people who do not meet their relatedness needs, use Facebook as a coping strategy. In addition, they found a positive correlation between Facebook and connection in which Facebook usage acts as a rewarding experience by which people get relatedness.

Another perspective presented in the literature is based on two opposing hypotheses: the stimulation and displacement hypothesis (Valkenburg & Peter, 2007). The stimulation hypothesis specifies that SNS usage increases for those people who have difficulties in creating social relationships and therefore, this usage may be beneficial in increasing well-being, reducing loneliness, and becoming more connected. Some studies support this hypothesis (e.g., Valkenburg & Peter, 2007). However, results in the literature are mixed and there are also studies that support the displacement hypothesis. The displacement hypothesis proposes that time spent in SNS displaces time spent in face-to-face interactions (Nie & Erbring, 2002), which will consequently result in the disconnection of the individuals and not meeting their relatedness need.

The third theory that has attention in the literature is the Interpersonal-Connection Behaviors Framework (Clark et al., 2018). This framework advocates that SNS usage compromises the individual’s well-being when this usage is determined by behaviors that do not fulfil needs for acceptance and belonging (e.g., social comparison and isolation) and consequently do not fulfil the need of relatedness. On the contrary, SNS usage is beneficial for well-being when behaviours that satisfy needs of belonging and connectedness take place. All these theoretical perspectives in general terms seem to be reconciled in the explanation of the bidirectional outcomes obtained using SNS. Although it is outside the scope of this study to test bidirectional relationships, it does cover their common factor (i.e., relatedness) explicitly. This was achieved through integrating the use of SNS with loneliness and perceived social support, which are two constructs with implications for relatedness (Bolger, & Amarel, 2007; Durkel-Schetter, & Bennett, 1990).

In the context of the SDT theoretical framework, loneliness has been identified as a trigger for frustration stemming from the need for relatedness (Chen et al., 2021). Therefore, the question of whether SNS usage facilitates relatedness needs and satisfaction, or on the contrary causes more isolation (Pertegal et al., 2019), might be resolved through understanding the relationships between SNS usage, loneliness, and perceived social support. Based on this theoretically contextualised approach, this study hypothesizes direct and indirect effects between these three variables.

### Current Research

The aim of this study was to assess the mediation model of the relationship between SNS use, perceived social support from family (Fam) and perceived social support from friends-significant others (Fri-SO), with the mediating role of loneliness, in a sample of Spanish university students. The focus on students was appropriate because of the high frequency of SNS use in this population (Duggan et al., 2015). Additionally, as mentioned previously, loneliness and lack of perceived social support can be critical during this period, when students leave home of origin or school to face new challenges (Arnett et al., 2014). The current study will fill the gap in knowledge not addressed by prior studies as it will consider loneliness as a mediator. Given that SNS such as Instagram and WhatsApp are common ways for young people to interact with others, it is necessary to examine the relationships between these variables and outcomes related to their use. To fill the gap in this area of research it is important to highlight awareness of the implications of SNS usage for social relationships and personal wellbeing. Moreover, this study will include a sample from an occidental culture, contributing knowledge in this area of research, as most of the studies reported in the literature are focused on Asian samples, collectivist cultures (Huang, 2017). More culturally diverse research is needed, as cultural research in psychology has shown that norms for social support seeking differ across cultures (Liu et al., 2018). In addition to these, the present study is different from previous studies in that it examines more than one SNS, namely Instagram and WhatsApp. Moreover, the study focuses on inferred causal relationships between SNS use and perceived social support with the mediating role of loneliness. Furthermore, the theoretical rationale for examining perceived social support from two different sources comes from the fact that SNS users are connected with kin and non-kin social contacts (Bakshy et al., 2012). Based on previous literature and the theoretical perspectives presented, we predict that:

- Hypothesis 1. SNS will significantly associate with loneliness and perceived social support.
- Hypothesis 2. Loneliness will mediate the relationship between SNS and perceived social support. The path from loneliness to perceived social support will be negative. The hypothesis is formulated in this direction because higher feelings of loneliness are related to lower perceived social support (Cacioppo et al., 2006; Harrison et al., 2021b; Zhou et al., 2008).
- Hypothesis 3. SNS usage and loneliness will be associated differently with each source of perceived social support: from friends-significant others and family.

## Method

### Participants and Procedure

**Fig. 1:**
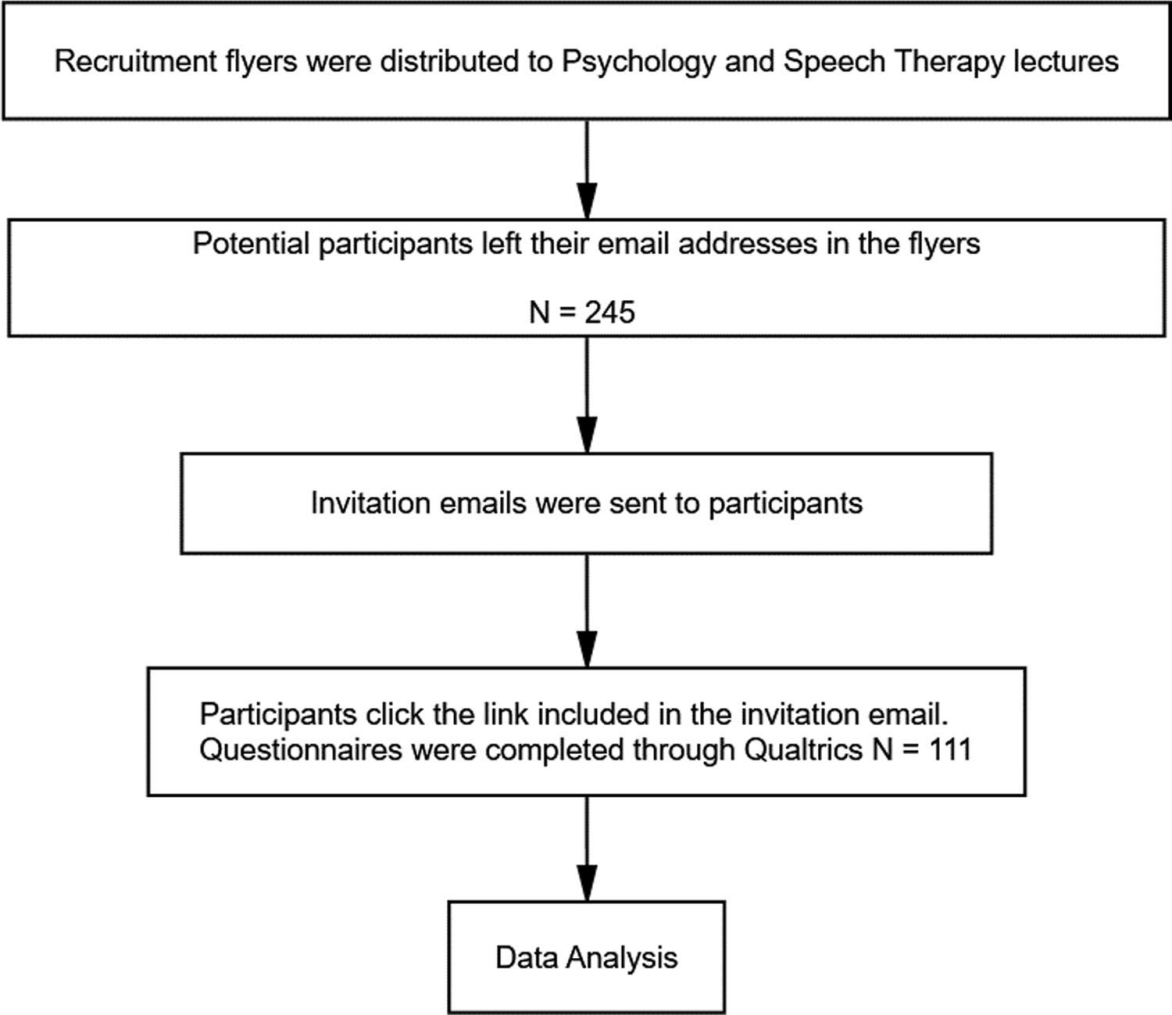
Flow chart on data collection process

Participants were required to be university students aged 18 or older. Both users and non-users of several digital technologies, new applications and SNS were invited to participate. In this study, a cross-sectional, quantitative design was used. The predictor variable is SNS usage, and the outcome variable is perceived social support (Fri-SO and Fam). Moreover, the mediator variable is loneliness, and the covariate is age. A total of *N*=111 Spanish participants from the University of Granada (Spain) were recruited by convenience sampling and responded to the online survey, (*M*_Age_ = 21.03, *SD*_Age_= 4.617; 88.3% female, 11.7% male). Figure 1 illustrates the data collection process. Of these participants, 92 (83%) were Psychology students and the remaining 19 participants (17%) were Speech Therapy students, and all were compensated with extra credit for their participation. Questionnaires were completed online through Qualtrics.com.

### Measures

#### Perceived Social Support

Perceived social support was measured by the 12 items of the Spanish *version* of the Multidimensional Scale of Perceived Social Support (Zimet et al., 1988). The MSPSS measures the extent to which an individual perceives social support from three sources: Friends (Fri) (Items 6, 7, 9, and 12) (α = 0.87), Significant others (SO) (Items 1, 2, 5, and 10) (α = 0.80), and Family (Fam) (Items 3, 4, 8, and 11) (α = 0.85). For example, “*I get the emotional help and support I need from my family*”, and “*My friends really try to help me*”). The items are rated on a seven-point Likert-type scale with scores ranging from ‘very strongly disagree’ (1) to ‘very strongly agree’ (7). The subscales of friends and significant others were merged into a single factor (Fri-SO) based on good fit indices, factor loadings, and for parsimony. This finding is congruent with previous studies that also combined the items of friends and significant others into a single factor with samples of Chinese adolescents and young adults (e.g., Chou, 2000). For the current sample, the Cronbach’s α coefficient of Fam’ subscale was 0.92, and the Fri-SO was 0.90.

#### Loneliness

Loneliness was assessed using the 20 items revised UCLA Loneliness Scale (Russell, 1996) in its Spanish version (e.g., “How often do you feel that there is no one you can turn to?”). The original version of the scale has a coefficient α ranging from 0.89 to 0.94. Participants were asked to indicate their responses on a 4-point Likert type scale (1 = *never*, 4 = *always*), with higher scores indicating higher levels of loneliness. For the current sample, the Cronbach’s α coefficient was 0.92.

#### SNS Usage

SNS usage was measured through the frequency assessment of Instagram, WhatsApp use, and Facebook (although the latter was not included in the latent variable as a measurement component for not meeting with essential requirements). The items were introduced by “Please indicate how often you use each of the following social networks and applications”. Moreover, the three items were rated with the 10-item frequency response scale created by Rosen et al. (2013), which includes: never, once a month, several times a month, once a week, several times a week, once a day, several times a day, once an hour, several times an hour, and all the time. For the current sample, the Cronbach’s α coefficient was 0.48 with the three items, and 0.52 if Facebook was deleted.

## Analysis

All data were explored and screened through descriptive statistics. Data were also tested for reliability by Cronbach’s Alpha and normality through skewness and kurtosis. Relationships between variables were tested through bivariate correlations. After this, a measurement model was conducted to test the factor structure of SNS as a latent variable. This latent variable was formed by two measurement components: Instagram and WhatsApp. Initially, Facebook was included in the latent variable as a measurement component, however, the factor loading (0.24) did not support its inclusion. Then, the relationships between use of SNS, loneliness, perceived social support from family and perceived social support from friends-significant others were modelled to test their relationships with each other. The model fit was assessed with the consultation of a range of the more reliable fit indices (Hu & Bentler, 1999), namely, relative chi-square statistic (χ2/df), the Root Mean Square Error of Approximation (RMSEA), Comparative Fit Index (CFI), Tucker-Lewis Index (TLI) and Standardized Root Mean Squared Residual (SRMR). Models are considered to fit the data adequately at values of χ2/df ≤ 2 to 3, ≤.08 for the RMSEA (Browne & Cudeck, 1993), ≥.90 for the CFI and TLI, (Bentler & Bonett, 1980) with values above .95 preferred and values ≤.08 preferred for SRMR.

A critical proceeding in structural equation modelling (SEM) is setting an appropriate sample size, although there is no consensus in the literature regarding what would be a sufficient sample size (Wang & Wang, 2012). Nevertheless, usually the minimum sample size for conducting SEM has been considered as *N* = 100-150 (Anderson & Gerbing, 1988; Ding et al., 1995; Tabachnick & Fidell, 2007; Tinsley & Tinsley, 1987). In addition, evidence exists that even with quite small sample sizes, SEM models could be meaningfully tested (Hoyle, 1999).

## Results

All the variables present good reliabilities (see Table 1) except SNS, which has poor internal consistency according to the criterion (≥ 0.7) (George & Mallery, 2010). However, the scale for SNS consists of two short items, which can lead to low alpha (Tavakol & Dennick, 2011). In terms of the use frequency of the three types of SNS, WhatsApp was the most used (*M=* 8.91*, SD=* 1.17*),* followed by Instagram *(M=* 6.66*, SD=* 2.85*) and Facebook (M=* 4.45*, SD=* 2.74*).*

**Table 1:** Correlation matrix, descriptive statistics and reliabilities for variables used in SEM.

	(1)	(2)	(3)	(4)	(5)
Age (1)					
SNS (2)	-.42**	1			
Lone (3)	.12	-.26**	1		
Fri-SO (4)	-.13	.33**	-.63**	1	
Fam (5)	-.19*	.20*	-.28**	.26**	1
Mean	21.03	15.57	41.24	5.40	5.28
Midpoint	-	11	50	32	16
SD	4.68	3.59	.31	1.13	.75
α	-	.52	.92	.90	.92
Skew	-	-.92	.60	-.69	.92
Kurto	-	.01	-.24	.12	2.22

Key: Skew = Skewness; Kurto = Kurtosis; Lone = Loneliness; SNS = Social Networking Sites; Fri-SO = Perceived social support from friends-significant others; Fam = Perceived social support from family. * =p ≤ .05; **=p ≤ .01.

The skewness and kurtosis absolute value of each variable were within the range of ± 1.96, except for a value of 2.22 in kurtosis, indicating only a small departure from normality in the case of perceived social support from family. Nevertheless, when using SEM, the values of skewness and kurtosis for normal distribution data are respectively below ±3 and ±10 (Brown, 2015).

The two sources of perceived social support, friends-significant others and family are significantly related to SNS use and loneliness. However, perceived social support from friends-significant others has a stronger relationship with the two than perceived social support from family. Also, the direction is negative in relation to loneliness. Perceived social support from family and friends-significant others are significantly but not highly related to each other (*r*=0.26, *p*<.01). This weak association justifies treating them as separate outcomes. Finally, there is a correlation between SNS and age, suggesting that as individuals get older, they may be less likely to use SNS (r= -0.42), although as noted, this does not include Facebook.

### Structural Equation Modeling

**Fig. 2:**
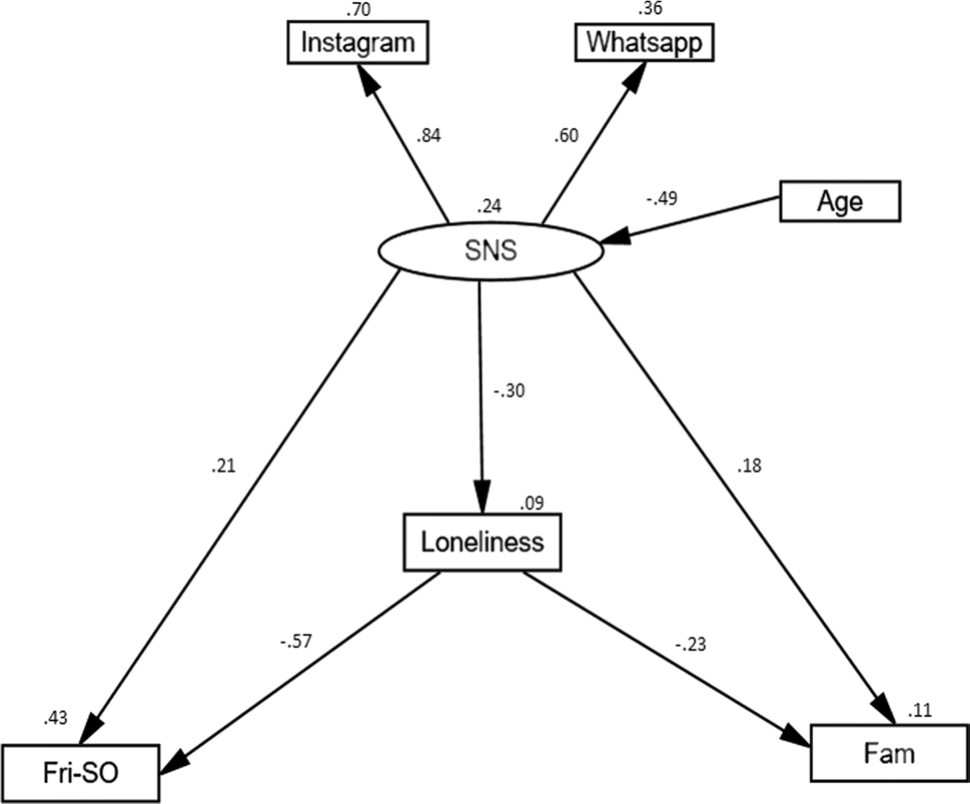
SEM for Social Networking Sites, loneliness, perceived social support from friends-significant others, perceived social support from family and age

The model presented in Fig. 2 was constructed by means of testing the relationships between SNS, use to loneliness, perceived social support from friends-significant others and from family, in Spanish university students, using Structural Equation Modeling (SEM). Before analysis, a measurement model was used to test the factor structure of SNS as a latent variable. This finally consisted of two measurement components: Instagram and WhatsApp. The factor loadings (≥ .4) (Hair Jr et al., 2010; Stevens, 2009) supported a one-factor solution. Initially, Facebook was included in the latent variable (SNS) as a measurement component with the other two mentioned previously. However, the factor loading (.24) did not support the inclusion of this item and was consequently removed.

The significant direct and indirect effects with beta coefficients are shown in Table 2. Results indicated that use of SNS, was positively and significantly related by direct effects to perceived social support from friends-significant others (β = .21, *p* ≤ .05), but was not significantly related to perceived social support from family (β = .18, *p* > .05). In contrast, loneliness was negatively related to the two social support subscales: respectively, (β = -.57, *p* < .01 & β = -.23, *p* ≤ .05). Moreover, when mediating the relationship between use of SNS and perceived social support from family, loneliness (β = .07) was not statistically significant as observed from Bootstrapping (95% Confidence Intervals [CI]: -.02, .15, *p* > .05). However, loneliness mediated the relationship between SNS and perceived social support from friends-significant others (β = .17) [CI]: .04, .30, *p* ≤ .01).

**Table 2:** Standardized Effects for the path model presented in Fig.1.

Causal Variable	Endogenous Variable			
	Fri-SO	Fam	Loneliness	SNS
SNS Direct Effect Indirect Effect (CIs 95%) Total Effect				
	.21*	.18	-.30*	-
	.17** (.04 to .30)	.07 (-.02 to .15)	-	-
	.38**	.25	-.30*	-
Loneliness Direct Effect Indirect Effect (CIs 95%) Total Effect				
	-.57**	-.23**	-	-
	-	-	-	-
	-.57**	-.23**	-	-
Age Direct Effect Indirect Effect (CIs 95%) Total Effect				
	-	-	-	-.49**
	-.18** (-.38 to -.05)	-.12 (-.37 to .01)	.15** (.05 to .40)	-
	-.18**	-.12	.15**	-.49**

*CIs= confidence intervals (95% upper and lower boundaries).*

*p ≤ 0.05, **p ≤ 0.01.

In addition, age was directly and negatively related to SNS (β = -.49, *p* ≤ .01). Also, age had significant indirect effects on loneliness (β = .15) [CI]: .05, .40, *p* ≤ .01), and perceived social support from friends-significant others (β = -.18) [CI]: -.38, -.05, *p* ≤ .01), but not significant indirect effects on perceived social support from family (β = -.12) [CI]: -.37, .01, *p* > .05).

The overall model was a good fit (χ2(7) = 3.54, RMSEA=.000, CFI=1, TLI=1.06, SRMR=.0262).

Instagram and WhatsApp demonstrate strong factor loadings onto SNS, .84 and .60, respectively. In addition, age has a moderate, negative association with SNS (β = -.49) and accounts for 24% of variance. Moreover, SNS is systematically related to perceived social support from friends-significant others (positively), perceived social support from family (positively) and loneliness (negatively). Variance accounted for on these variables (respectively) by SNS is 43%, 11% and 9%. Furthermore, it can be seen from Table 2 that the indirect effect of SNS through loneliness is statistically significant with reference to perceived social support from friends-significant others but not to perceived social support from family.

## Discussion

The main purpose of this study was to assess the relationship between SNS usage, and perceived social support from two different sources: friends (including significant others) and family, with the mediating role of loneliness, in a sample of Spanish university students. To the best of our knowledge, the current research is one of the first studies to date to clarify the mediating mechanisms of loneliness in the association between SNS use and perceived social support from the two different sources. The predictor variable (or independent variable) is SNS, which is a measurement model. This variable predicts the three variables below it in Fig. 1 (loneliness, and perceived social support from friends-significant others and family) by three direct effects and two indirect effects. However, SNS also serves as an outcome or dependent variable because the variable ‘age’ explains 24% if the variance on it. SNS is clearly central in this model as it is both an independent and dependant variable (it has a dual function). The measurement model, SNS, is formed by Instagram and WhatsApp. This is a contribution in this area of research as most of the studies found in the literature are focused only on Facebook (Meng et al., 2017).

The results revealed that: (a) SNS usage was positively related by direct effects to perceived social support from friends-significant others but was not significantly related to perceived social support from family; (b) loneliness acted as a significant mediator of the relationship between SNS and perceived social support from friends-significant others but was not a significant mediator of the relationship between SNS and perceived social support from family; (c) age was directly related to SNS negatively and had significant indirect effects on loneliness and perceived social support from friends-significant others.

### SNS Usage Direct Effects to Perceived Social Support from Friends-Significant Others

The results found in this study suggest that the implications of SNS usage are not the same for all the sources of perceived social support. In this respect, it seems that SNS usage is related to perceived social support from friends-significant others while it is not related to perceived social support from family. Thus, participants who reported use of SNS more frequently also reported higher levels of perceived social support from friends-significant others. This finding could be because the online interactions with “family” are less frequent compared with online interactions with “friends” and “significant others”. In addition, this positive perception could also be explained by a sense of belonging. A study conducted by Wong et al. (2019) found that a greater desire to belong significantly and positively predicted the frequency of Instagram usage, as well as perceived social support from friends and significant others. Furthermore, a study conducted by Oh et al. (2014) found a non-significant relationship between frequency of SNS usage and perceived social support. However, they considered three dimensions of perceived social support (companionship, esteem, and appraisal). Thus, to our knowledge, different sources of perceived social support were not considered previously in research examining the relationships between use of SNS, loneliness and perceived social support. Regarding the results obtained, the differentiation of these two types of support could have important practical implications. For instance, if university students lack perceived social support from friends, this can be enhanced with the use of SNS. Nevertheless, if students lack perceived social support from family, perhaps other interventions are more appropriate.

### Mediating Role of Loneliness

Another important finding is the mediating role of loneliness. A study conducted by Lin et al. (2020) found a relationship between an active use of SNS and loneliness, with the mediating role of social support. However, how the use of SNS is related to perceived social support considering loneliness as a mediator in this relationship was not examined previously in the literature. This paper found that Loneliness acted as a mediator in the relationship between SNS usage and perceived social support from friends-significant others, suggesting that SNS may benefit users by increases in perceived social support, through decreases in felt loneliness. This finding is congruent with the findings of multiple other studies that have reported that increases in SNS use facilitates connection (Ahn & Shin, 2013), and therefore satisfies the need of relatedness. A study conducted by Pittman and Reich (2016) found that social media platforms that are based only on images, such as Instagram, decreased feelings of loneliness because they provide enhanced intimacy and closeness. Differences in the activities of SNS usage could explain the inconsistent results that have been found in the research about the relationship between SNS usage and loneliness (Wang et al., 2018b).

Furthermore, the findings from this study can be supported by the need for relatedness within Self-Determination Theory (Ryan & Deci, 2000). Moreover, other supporting theories that share this need as a common factor include the Interpersonal-Connection Behaviors Framework (Clark et al., 2018) and the stimulation hypothesis (Valkenburg & Peter, 2007). These results suggest that the use of SNS contributes to interpersonal connection, through the decrease in loneliness and increase of perceived social support. The outcomes seem to indicate that this usage was driven by the quest for connection, acceptance and belonging. Therefore, congruent with previous research, the positive or negative consequences of using social network sites would depend on the nature of that use (Clark et al., 2018; Phu & Gow, 2019).

Findings are likely to be useful for administrators, and policy makers in Higher Education as the understanding around various types of social support may assist in tailoring specific and appropriate supportive interventions. The remediation of loneliness might be advantageous for retention, progression, and integration into the academic community in Higher Education.

### The Effect of Age

It was not a primary goal of this study to explore these relationships in different age groups. However, results showed that age was directly related to SNS negatively and had significant indirect effects on loneliness and perceived social support from friends-significant others. Results indicated that older participants used SNS less frequently. In addition, age had an indirect effect on loneliness by SNS usage. This means that older participants felt higher levels of loneliness, possibly exacerbated through SNS usage. This finding may indicate that young adults are more involved in connecting to their SNS and interacting with their peers via Instagram or WhatsApp. However, older students, who perhaps are more used to face-to-face interactions than the younger students, through using their SNS, may experience negative outcomes (e.g., loneliness and isolation) in a greater way. In addition, the older the students the lower perceived social support from friends-significant others through SNS usage. Contrary to this finding, in the meta-analysis conducted by Liu et al. (2018) it was reported that the relationship between SNS and social support was stronger for older students (undergraduates and graduates). This may suggest that older students are more responsive online and are better at completing the task of social support transactions. Nevertheless, results from the study conducted by Liu et al. (2018) consider various types of social support, which differs considerably to the type addressed in the present study, namely perceived social support from family and friends-significant others.

### Implications for Theory and Practice

The mixed findings in the literature could be in part due to cultural differences. The current study contributes with knowledge to the literature as most of the studies are focused on Asian samples (collectivistic culture) and this study is focused on a Western European sample (individualistic culture). The literature suggests that Asian SNS users might receive more offline social support from their SNS usage, which also leads to stronger relationships between SNS and perceived social support than in Western cultures (Liu et al., 2018). However, more research focused on Western cultures is needed. Therefore, knowledge of the relationship between SNS usage and perceived social support considering different age groups and cultures can help to design better intervention programs adapted to the target audience (Liu et al., 2018).

Initially, Facebook frequency of usage was assessed in this study, however, its inclusion in the model was withdrawn due to a low factor loading. The reason behind this finding could be related to the result found by the survey research data conducted by the Pew Research Center (2018) in which 42 percent of Facebook users had disengaged from Facebook in the previous year. Indeed, the mean of Facebook usage frequency in the present study was lower than the other two (*M*_*Facebook*_*= 4.45, M*_*WhatsApp*_*= 8.91 and M*_*Instagram*_*= 6.66).* Therefore, this study reflects recent changes with the focus on Instagram and WhatsApp as previous work has mainly focused on Facebook. Moreover, the present study highlights the importance of looking at specific sources of social support, loneliness, and age for a better understanding of the effects of SNS usage. This step may help to develop more conscious and adaptive uses of technology as key enablers in the future to address loneliness and isolation, consequently countering the negative impact they may have on overall health and wellbeing.

A strength of this study is that it is embedded within the context of several theoretical perspectives. For example, Self-Determination Theory by Deci and Ryan (Ryan & Deci, 2000). This conceptual framework captures the words within the acronym “CAR” - Competence, Autonomy and Relatedness. Participants in SNS platforms may feel confident in their Competence to use the platforms (e.g., responding with wit and using multimedia to communicate). They may also feel quite independent in expressing their individuality and in putting their own unique brand (e.g., humour) on what they do - i.e., Autonomy. Perhaps the real strength of this theoretical model is in Relatedness as this gives users such as students the opportunity to connect with others and feel a sense of community and belonging as part of the in-group. In contrast they may be left feeling excluded and isolated. This study has embraced these aspects of the model with attention to loneliness and social support.

The second theoretical context addresses the two opposing hypotheses: the stimulation and displacement hypothesis (Valkenburg & Peter, 2007). The stimulation hypothesis predicts that SNS usage may be beneficial in increasing well-being, reducing loneliness, via a positive effect on social relationships. However, the displacement hypothesis predicts that time spent in SNS displaces time spent in face-to-face interactions (Nie & Erbring, 2002), which may consequently result in the disconnection of the individuals, not meeting their relatedness need, and having a detriment effect on their well-being levels. Results from this study show a mediating role of loneliness in the relationship between SNS usage and perceived social support from friends-significant others. Thus, results seem to be supporting the stimulation hypothesis, because this is suggesting an association between higher SNS usage, lower feelings of loneliness, and higher perceived social support. However, a longitudinal study would be required to test causality.

The third theory that resonates with this study is the Interpersonal Connection Behaviours Framework (Clark et al., 2018). As implied by the name, repeated behaviours that link individuals through dynamic and repeated interpersonal activities may lead to connectivity and relatedness. However, through SNS usage this may work in a contrary direction in a way that might compromise an individual’s wellbeing so that they do not find fulfilment for their social needs through acceptance and belonging. Their SNS usage may thus impair the quality of their personal wellbeing. In contrast, a well-regulated and sensibly balanced use of SNS may be a contributing factor in optimising student wellbeing. Moreover, during the pandemic this was an essential outlet for students.

These theoretical perspectives provided a good foundation for the current research in terms of both positive and negative outcomes. For example, based on the Self Determination Theory conceptual framework, loneliness blended in as the opposite of relatedness (Chen et al., 2021). In the same vein, social support naturally weaves into these theoretical frameworks as it is seen as adaptive to wellbeing and a buffer for negative outcomes (Lo, 2019).

### Limitations and Future Research Directions

Although the direction of causation is inconclusive, model fit, and parameter estimates suggest that future research should consider the implications of loneliness and age in the context of SNS usage in optimizing perceived social support from friends-significant others. Furthermore, future work should also test the longitudinal relationships between SNS usage, loneliness, and perceived social support. This would address the question of whether these relationships are maintained in the long-term. Moreover, it would be interesting to differentiate SNS-related perceived social support compared to regular (offline) perceived social support to compare the quality of both.

Future work could also look at the totality of the student experience with reference to a full skill set and the development of academic and personal competencies. For example, Taghani and Razavi (2021) in a recent study concluded that teaching study skills to students will lead to improvement in self-efficacy, academic engagement, and academic performance. They emphasised that positive experiences stem from success and this further enhances self-efficacy. In addition, Asarta and Schmidt (2017), and Razavi (2021) concluded that academic performance empowers students to reflect on and recount on their learning content and processes over the past year. According to Partovi and Razavi (2019), issues related to students’ personal discipline and commitment also come into play and these researchers accentuate regular attendance at learning sessions. This broader perspective will facilitate mapping out the ongoing pathway to continued success. Therefore, for the present study to be enhanced and enlarged, the context of the full student experience should be incorporated in future research.

The present study is not without limitations. Firstly, the use of self-reports could have resulted in socially desirable answers. Secondly, this study, like most studies in this area, is cross-sectional and relies on a student sample. Thus, generalizing the results to different populations must be undertaken with caution. These concerns can be counter-balanced, however, by the validity statistics presented, by the variances extracted and the effect sizes elicited through the constructs. In addition, the use of SEM provides an approach for thinking about a causal structure from SNS usage to loneliness and consequently from loneliness to perceived social support from friends and significant others. The associations in the model suggest genuine causal effects that could be empirically tested in future studies. For instance, frequency of SNS usage

could be manipulated by asking participants to reduce or even stop using their accounts for a limited period, while a comparator/control group would continue using their account as normal. Levels of loneliness and perceived social support could be measured before and after that period.

Many online social support networks have been set up for people with particular problems such as bereavement, divorce, chronic and acute illnesses, and mental health problems (Kaplan et al., 2011). Positive outcomes may result from this usage as individuals within these groups may feel a stronger sense of camaraderie and support from other individuals with similar problems. It would be interesting to ascertain whether individuals who have specific problems are more likely to move away from mainstream SNS toward sites where acceptance and connection may be more likely. Moreover, positive outcomes might be obtained through the usage of SNS through the quarantine or the social isolation experienced during the COVID-19 pandemic. Besides, a study conducted by Masciantonio et al., (2021) found that active usage of Instagram was positively related to satisfaction with life. In addition, another finding was that through social support, Instagram usage was positively related to negative affect. Furthermore, they found a positive relationship between Twitter and satisfaction with life through social support; and passive usage was positively associated with negative affect through upward social comparison. Literature shows that positive outcomes or potential negative outcomes result depending on the context of the usage and the SNS type, and therefore, researchers need to take these factors into account.

Finally, although this study covers the relatedness aspect of Ryan and Deci’s (2000) Self-Determination Theory, implying the search for connection and possibly online community, the other two aspects of the theory are not explicitly covered. However, the aspect of autonomy might be implied when SNS usage is driven by self-determined and autonomy-enhancing behaviours or on the contrary by autonomy-impeding behaviours such as fears (Vorderer et al., 2018). SNS users may be either high or low on autonomy: high if they use online engagement to reduce face-to-face contact; low if engagement is designed to elicit the endorsement of others. For competence, individuals may feel equal to others by sharing photos, messages with witty replies, and imaginative suggestions etc. In addition, the aspect of competence could be explained in a future study that covers competence perceptions such as in digital self-efficacy beliefs. Additional investigations are crucial to understand how the use of SNS is related to perceived social support. Social support researchers have shown that characteristics of the person who is receiving the social support could be determined by how they perceive that support (Collins & Feeney, 2004). Therefore, future studies could examine how the social orientation tendency, which includes three types of social orientation: prosocial, individual, and competitor (Lewis & Willer, 2017), affects the relationship between use of SNS and perceived social support.

## Conclusions

Based on the common factor of the SDT, the stimulation-displacement hypothesis, and the interpersonal-connection behaviours framework, which is relatedness, the present study examined the direct and indirect effects between SNS usage, loneliness, and different sources of perceived social support. Accordingly, the current study has extended previous research findings in showing that loneliness and age are important covariates in assessing the relationship between use of social network sites and perceived social support from friends-significant others. This study has important implications for outlining that SNS usage may benefit college students by increases in perceived social support from friends-significant others, via decreases in felt loneliness. Moreover, it also outlines that loneliness could increase through SNS usage in older college students. In a world of constant connectivity but with high levels of loneliness, which has faced isolation and lockdown periods due to the Covid-19 crisis (Saltzman et al., 2020), the findings from this study are of importance for those who develop interventions and public policy surrounding a healthy and adaptive usage of technology and a decrease of loneliness’ feelings.
